# Viral Infection Affects Sucrose Responsiveness and Homing Ability of Forager Honey Bees*, Apis mellifera* L.

**DOI:** 10.1371/journal.pone.0077354

**Published:** 2013-10-10

**Authors:** Zhiguo Li, Yanping Chen, Shaowu Zhang, Shenglu Chen, Wenfeng Li, Limin Yan, Liangen Shi, Lyman Wu, Alex Sohr, Songkun Su

**Affiliations:** 1 College of Animal Sciences, Zhejiang University, Hangzhou, Zhejiang, China; 2 USDA, ARS Bee Research Laboratory, Beltsville, Maryland, United States of America; 3 ARC Centre of Excellence in Vision Science, Research School of Biology, College of Medicine, Biology and Environment, the Australian National University, Canberra, Australia; 4 College of Computer, Math, and Natural Sciences, University of Maryland, College Park, Maryland, United States of America; 5 College of Bee Science, Fujian Agriculture and Forestry University, Fuzhou, Fujian, China; Monash University, Australia

## Abstract

Honey bee health is mainly affected by *Varroa destructor*, viruses, *Nosema* spp., pesticide residues and poor nutrition. Interactions between these proposed factors may be responsible for the colony losses reported worldwide in recent years. In the present study, the effects of a honey bee virus, Israeli acute paralysis virus (IAPV), on the foraging behaviors and homing ability of European honey bees (*Apis mellifera* L.) were investigated based on proboscis extension response (PER) assays and radio frequency identification (RFID) systems. The pollen forager honey bees originated from colonies that had no detectable level of honey bee viruses and were manually inoculated with IAPV to induce the viral infection. The results showed that IAPV-inoculated honey bees were more responsive to low sucrose solutions compared to that of non-infected foragers. After two days of infection, around 10^7^ copies of IAPV were detected in the heads of these honey bees. The homing ability of IAPV-infected foragers was depressed significantly in comparison to the homing ability of uninfected foragers. The data provided evidence that IAPV infection in the heads may enable the virus to disorder foraging roles of honey bees and to interfere with brain functions that are responsible for learning, navigation, and orientation in the honey bees, thus, making honey bees have a lower response threshold to sucrose and lose their way back to the hive.

## Introduction

 Honey bees (*Apis mellifera* L.) play a vital role in global food production and economy [1]. However, honey bee colony losses in recent years have had a devastating effect on the agricultural industry and ecosystems that rely on honey bees for pollination. Colony Collapse Disorder (CCD) is a poorly understood phenomenon in which workers abruptly disappear and do not return to the hive but leave behind live queen and brood in the colony. This occurred during the winter of 2006-2007 in the US and colony losses reported in recent years in other parts of the world [2,3], poses a particular threat to apiculture and agriculture worldwide. A metagenomic survey of microflora in CCD hives showed that Israeli acute paralysis virus (IAPV), a virus that had not been previously reported in the US, displayed a strong correlation with CCD. IAPV was detected in 86% of CCD-affected colonies but in less than 5% in healthy colonies [4]. As a result, the disease mechanisms of IAPV infection in honey bees have been the subject of extensive research since then.

 IAPV is a member of the genus *Aparavirus*, a new class of viruses in the *Dicistroviridae* family [5]. IAPV was first reported in 2004 in Israel where IAPV infected honey bees were found with symptoms of shivering wings, paralysis, and then death outside of the hives. The severe bee mortality caused by IAPV led to heavy losses in Israeli apiculture [6]. Since its first detection, IAPV infection has been reported in many other parts of the world [7-10]. A new study showed that *Varroa destructor* [11], an ectoparasite of the honey bee, has been catastrophic for the beekeeping industry and is an effective vector of IAPV [12]. The number of copies of IAPV in honey bees was positively correlated with the density of *Varroa* mites and the time period of *Varroa* feeding. The association of IAPV and *Varroa* mites could cause increased damage to honey bees and poses a particular risk to bee heath. Recent study by Maori et al. (2009) and Hunter et al. (2010) offers some encouragement that the disease caused by IAPV can be abated by use of RNAi-based technology [13,14]. 

 Although data regarding IAPV infection in honey bees have been accumulated rapidly, little information is available so far about the effects of IAPV on honey bee behaviors. The present study elucidates the roles of IAPV on sucrose responsiveness and the homing ability of pollen foragers from the view of behavioral experiments based on proboscis extension response (PER) and radio frequency identification (RFID) systems. When the antenna of a worker bee is touched with a droplet of sucrose solution, she can extend her proboscis instinctively. Sucrose responsiveness of honey bees can therefore be measured by using PER [15]. A learning paradigm can be established between extension of the proboscis elicited by sucrose solution and odor [16]. The learning paradigm based on PER is employed to study the molecular mechanisms underlying learning and memory formation in honey bees [17]. The learning paradigm was also used to evaluate the effects of exogenous substances (imidacloprid, nicotine and caffeine, etc.) on learning performances of honey bees [18-20]. The PER paradigm was first employed by Iqbal and Mueller to evaluate the effects of Deformed wing virus (DWV) on sucrose responsiveness and learning performances of honey bees, which showed that DWV makes honey bees more responsive to low sucrose concentrations and results in detrimental effects on associative learning in honey bees [21]. The PER paradigm could be operated simply and reliably, but its consistency could be affected by genotype, age, foraging role, and sex [22-25]. Pollen foragers were, therefore, selected in the study in order to avoid these variable factors.

 Homing ability of honey bees mainly depends on their spatial memory and navigation [26,27], and could be impaired by pesticides and pathogens [28-30]. Kralj and Fuchs showed that *Varroa* mites affect flight duration and homing ability of infested foragers [29]. They found that *Nosema* sp. influence flight behaviors of infected foragers as well [30]. Information about the effects of viral infection on homing abilities of foragers was therefore investigated in the study using the RFID tagging technology. RFID systems, which were first used in honey bee research in 2003 [31], have been employed in research on honey bee behaviors and the honey bee decline due to pesticides in recent years [28,32-34]. Previous studies have demonstrated that the RFID system is a potent way to record changes in foraging behavior of honey bees after the administration of exogenous substances to the honey bees. By using the RFID system, the effects of IAPV on homing ability of honey bees could therefore be monitored effectively over a period of several days without intermission. Our results demonstrated unambiguously that IAPV affected the sucrose responsiveness and homing ability of infected foragers.

## Materials and Methods

### Ethics Statement

 All necessary permits were obtained for the described field studies. Honey bee colonies were used for field studies with the permission from beekeepers. Behavioral experiments were carried out at the Zhejiang University; therefore, no permits were required for the location. The European honey bee (*Apis mellifera* L.) used in the study is neither an endangered or protected species.

### Screening for healthy colonies

Honey bee colonies were selected from two apiaries located in Northern Zhejiang province, China. Honey bee colonies used in the study were screened by RT-PCR according to the methods described by the previous studies [8,35] for the presence of seven common honey bee viruses including Acute bee paralysis virus (ABPV) [36], Black queen cell virus (BQCV) [37], Chronic bee paralysis virus (CBPV) [38], DWV [39], IAPV [6], Kashmir bee virus (KBV) [40] and *Sacbrood virus* (SBV) [41]. Apparently healthy and strong colonies showed a relatively low level of honey bee viruses in comparison to weak colonies [12,42]. Twenty adult worker honey bees, twenty larvae, and thirty eggs from each strong candidate colony with about 12 frames were collected and examined for the presence or absence of the seven viruses [43,44]. Colonies that were identified without detectable viruses were selected in our subsequence studies. 

### Isolation and purification of IAPV

 IAPV was isolated according to the protocols kindly provided by Maori et al. [6]. Briefly, about 250 crawling honey bees were collected from an apiary infected with IAPV. These honey bees were homogenized with sand in a mortar in the presence of 0.01 M phosphate buffer contained 0.02% DETCA (pH 7.2, Sigma-Aldrich Cat #22,868-0). The homogenate was centrifuged at 800 rpm for 20 minutes and supernatant was collected and centrifuged at 40,000 rpm at 4 °C for 4 hours. The pellet was collected and thoroughly dissolved with 0.01 M phosphate buffer which contained 0.4% Sodium deoxycholate (Sangon Biotech, Shanghai, China) and 4% Brij 58 (Sangon Biotech, Shanghai, China). The solution was centrifuged at 800 rpm for 15 minutes. The supernatant, which was collected and mixed with CsCl (Sangon Biotech, Shanghai, China), was centrifuged at 40, 000 rpm at 18 °C for 24 hours. After centrifugation, the band was collected and dialyzed in dialysis membrane at 4 °C within double distilled water. A sample of 20 ul was then used for confirming the purity of IAPV by RT-PCR without contamination of other common honey bee viruses.

### Responsiveness to sucrose

 Pollen foragers from healthy colonies were caught during periods of peak foraging activity in the day between 8 and 9 a.m. at the hive entrance. A wire mesh screen was placed in front of the hive entrance to prevent honey bees entering the hive too quickly. Several 50 ml centrifuge tubes punched with small holes were used for capturing pollen foragers, with each tube collecting 1 or 2 pollen foragers. Pollen foragers were then immediately transferred from the centrifuge tubes into cages, and fed ad libitum with 50% (wt/wt) sugar water. The purified IAPV was diluted in phosphate-buffered saline (PBS: 137 mM NaCl; 3 mM KCl; 10 mM Na_2_HPO_4_; 2 mM KH_2_PO_4_, pH 7.2) with a ratio of 1: 200 and 1: 500 respectively. Honey bees were first immobilized in small glass vials individually by chilling on ice for about 3 minutes. Sixty honey bees from each group were injected with 1 ul of diluted IAPV or PBS by a 5 ul microsyringe (Hamilton^®^) between the fifth and sixth abdominal segment and honey bees with signs of hemolymph leakage after injection were discarded. Honey bees injected with PBS were designated as sham group. 

In order to test the sucrose responsiveness of pollen foragers using PER, IAPV, and sham, inoculated pollen foragers were mounted in small copper tubes individually. After a one hour restoration period in an incubator at 30°C in 75% humidity [15], honey bees were checked to ensure each honey bee could extend its proboscis freely. The antenna of the honey bees was touched with a droplet of the following concentrations of sucrose: 0.1, 0.3, 1, 3, 10, and 30% (wt/wt) according to methods described by previous studies [23,45]. After the stimulus of each sucrose solution, the antennae of honey bees were then touched with a droplet of water to avoid of possible sensitization due to repeated stimulus [23,45]. The stimulus was applied at 2-minute intervals evenly according to the previously described methods [15,21]. Honey bees that exhibited small movements of the proboscis (not extending its proboscis fully) during PER test were scored negative [45]. After the test, honey bees were tested by 50% (wt/wt) sugar water and any honey bee showing dull responses or not responding to 50% (wt/wt) sugar water was not used for data collection [46], and was collected for further analysis of the detection of IAPV in the study.

In the evening (5-6 p.m.), honey bees were fed one after another repeatedly with 50% (wt/wt) sugar water using a 20 ul pipette until they didn’t extend their proboscis reliably and quickly when their antennae were touched with 50% (wt/wt) sugar water [47]. After feeding, these honey bees were placed in an incubator (30 °C, 75%) overnight. The next morning (8 a.m.), honey bees were taken out of the incubator and placed at room temperature (24-26 °C). The honey bees were checked again to see if they could extend their proboscis freely and the PER test was conducted again. For honey bees injected with 1 ul of a 1: 500 dilution of IAPV, the number of honey bees responding to sucrose stimulus was observed and recorded at days 0, 1, 2 and 3 post-injection. For honey bees injected with 1 ul IAPV with a 1: 200 dilution, the number of honey bees responding to the sucrose stimulus was observed and recorded at days 0, 1 and 2 after injection.

### Quantification of IAPV by Real-Time qPCR

Total RNA was extracted from pooled heads of 3 foragers, and cDNA was generated from 1 ug of total RNA using PrimeScript^®^ RT reagent Kit (TaKaRa, Dalian, China) following the manufacturer’s instructions. The qPCR assay was conducted in a final total volume of 20 ul containing 10 ul 2x SYBR^®^ Premix Ex Taq^TM^, 2 ul 1:5 diluted cDNA, 1 ul sense primer (10 uM), 1 ul antisense primer (10 uM) and 6 ul sterile water in the Eppendorf Mastercycle^®^ ep realplex. Each PCR amplification was performed in triplicate, and no-template controls (NTC) were also run in parallel for each assay. The reaction mixtures were first denatured at 95 °C for 30 s, then 40 cycles of 95 °C for 5 s, 56 °C for 30 s, and 72 °C for 30 s, followed by a melt curve analysis. Also, a sample of 200 ul purified IAPV was used for RNA extraction and qPCR analysis to determine the exact copy number of IAPV of the purified virus solution. The primer sequences used for qPCR assay are listed in Table 1. 

**Table 1:** Primer sequences used for real-time RT-PCR, constructing and cloning IAPV-recombinant plasmids.

**Purpose**	**Primer sequence**	**Amplicon (bp)**	**Reference**
qPCR assay	F primer: 5’-GCCAGAGCAGGAAACGATGAC-3’	75	
	R primer: 5’-GGAGCGTGATTCGCCTTGTAG-3’		
Constructing recombinant plasmids	F primer: 5’-AGACACCAATCACGGACCTCAC-3’	475	[6]
	R primer: 5’-AGATTTGTCTGTCTCCCAGTGCACAT-3’		

 Purified IAPV specific amplicons were incorporated into the pMD^®^ 18-T vector (TaKaRa, Dalian, China) following the manufacturer’s protocol. Recombinant plasmid DNA was purified and the copy number of plasmid DNA was calculated based on the molar concentration and molecular mass of the recombinant plasmid consisting of plasmid vector and the PCR insert. The standard curve was established by amplifying the serially diluted plasmids in a qPCR assay. The sensitivity of the qPCR assay was determined by plotting the log of the initial quantities of 10 x serial diluted plasmid (10^1^ to 10^9^) against the corresponding threshold cycle (Ct) value. The amplification efficiency of each plasmid template was calculated from the slope of the standard curves according to the following formulas: E (%) = (10^(-1/slope)^-1) x 100. The exact copy number of IAPV load in heads of honey bee samples collected at day 0, day 1, and day 2 after injecting with 1 ul IAPV (1:200 dilutions) was determined by plotting Ct values of unknown samples to the established standard curve. 

### Assessment of survival rates

Before the homing experiments, a comparison of survival rates between honey bees injected with 1 ul PBS and honey bees injected with 1 ul IAPV with a 1:200 dilution was assessed. Pollen foragers were from four different colonies without detectable common viruses and there were 165 and 186 honey bees injected with PBS and IAPV respectively. After the injections, they were kept in cages at room temperature (24-26 °C) and supplied with a 50% sucrose and water solution. The proportion of live honey bees to dead honey bees was then calculated at 24 and 48 hours after injection.

### Homing experiments

**Figure 1:**
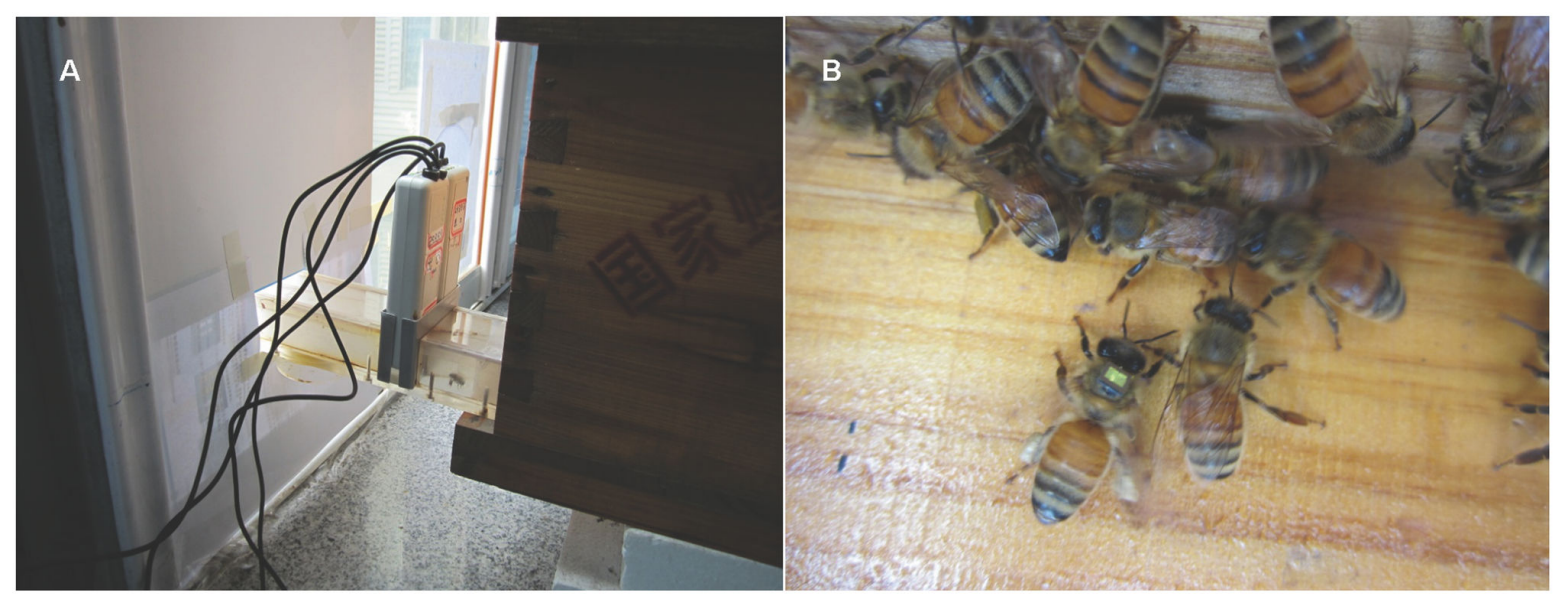
The RFID system used in homing experiment. (A) Two RFID readers were placed at the customized tunnel entrance of a nucleus hive with 3 frames. (B) A honey bee with the RFID tag glued to its thorax carrying pollen on its hind legs returns to the hive.

 The experiment was carried out from June - October, 2012. Three 3-frame nucleus hives consisting of approximately 4,000 honey bees were created from three original colonies and used in the homing experiment. The mic3-TAG passive 13.56 MHz tags stored a unique 64-bit number (approximately 2.4 mg, 2.0 x 1.6 mm) and readers (Microsensys 2k6 HEAD) were bought from Microsensys [32]. The ID numbers of tags were input into software running on a computer by a USB-Pen prior to being glued to the thoraxes of honey bees. A 30 cm long customized tunnel was linked to the entrance of a nucleus hive, and two readers were attached to the top of the tunnel (Figure 1). The software used for storing tag ID numbers and exporting data from the readers was designed by Sebastian Streit (“Beegroup ID2DB” © Beegroup, Sebastian Streit, 2003) [31]. 

 As the methods described above, honey bees were captured from the nucleus hives and were then immobilized on ice before injection. One group of 20 pollen foragers was injected with 1ul of IAPV with a 1: 200 dilution in PBS and another group of 20 pollen foragers was injected with 1 ul of PBS as a control. Each pollen forager was then equipped with an RFID tag on the thorax, using shellac glue, to identify the honey bee. Subsequently, the honey bees were restored in cages at room temperature (24-26 °C) and fed ad libitum with 50% (wt/wt) sucrose solution and water for an hour. After that, the honey bees were then placed in a black box and transported 500 meters away from the hive, at which point we then released these honey bees. Honey bees that could not take off within five minutes were discarded from the study [32]. The two readers recorded the entering or leaving of pollen foragers each day after the start of the experiment [32]. We did not stop observing returning honey bees until the last honey bee didn’t return to the hive for each individual trial. The homing experiment was carried out in three different nucleus colonies and each homing experiment was repeated three times for each nucleus colony. 

### Data analysis

 The percentage of honey bees showing PER was calculated by the number of honey bees showing PER divided by the total number of honey bees used for data collection each day after the treatment. Differences in responsiveness to sucrose solution between honey bees injected with IAPV and the control group was analyzed by the Fisher exact test. Comparison of survival rates between the two groups of honey bees was analyzed by independent samples t-test (SPSS Statistics 13.0, SPSS Inc., Chicago, IL, USA). Homing ability was assessed based on the number of bees not returning to the hive each day after the treatment. The data regarding homing ability was not normally distributed. Therefore, a non-parametrical Kruskal-Wallis test followed by Dunn's multiple comparison test was used to analyze the difference of homing ability between the two groups of honey bees using the Graphpad Prism 5 (GraphPad Software Inc, San Diego, CA, USA). Differences were considered significant at p<0.05 for all statistic tests and all tests were two-tailed.

## Results

### Responsiveness of IAPV-infected honey bees to sucrose

**Figure 2:**
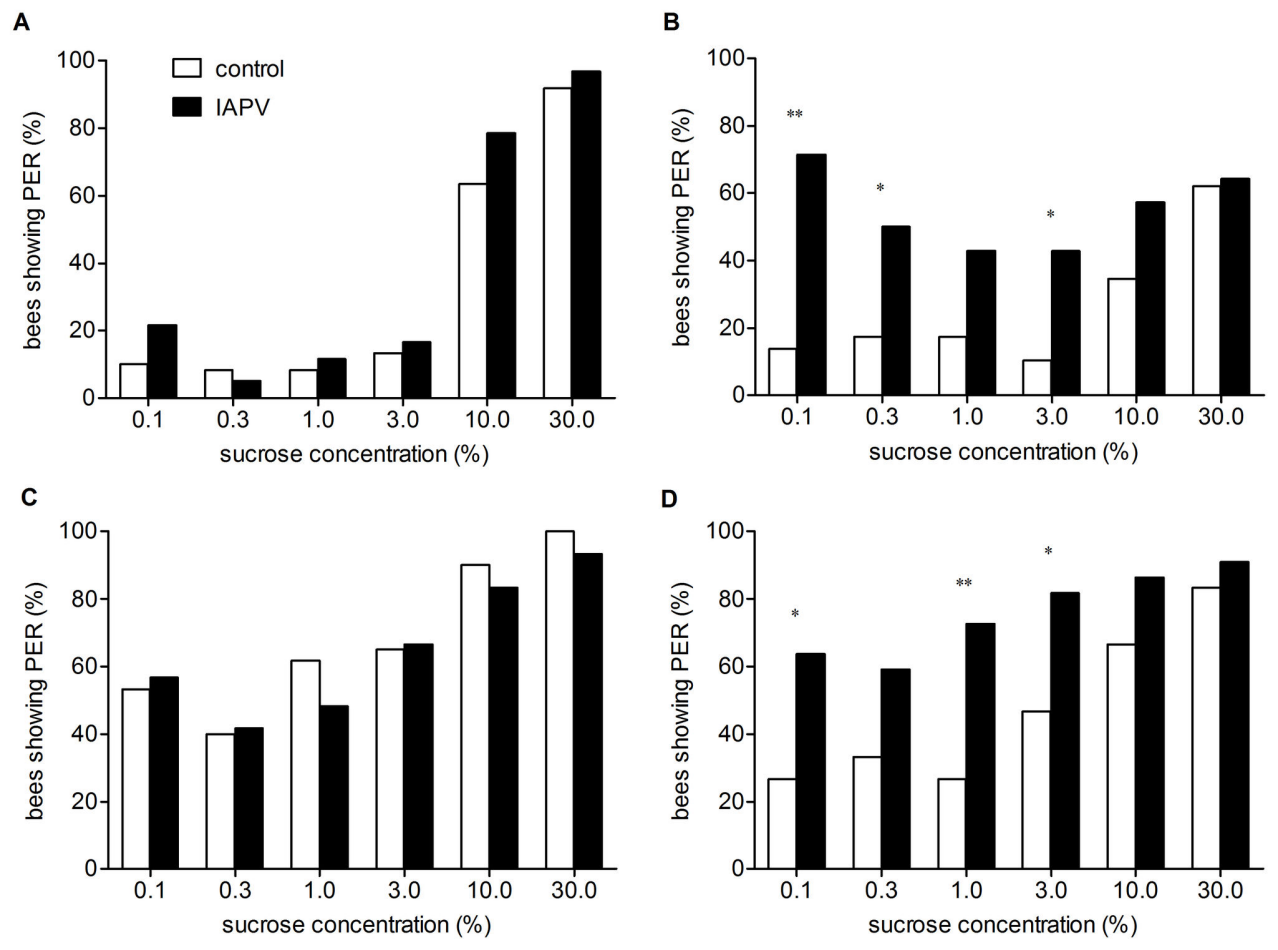
Comparisons between IAPV-injected honey bees and PBS-injected honey bees in their responsiveness to sucrose. Sucrose responsiveness of honey bees injected with IAPV with 1: 500 dilution and PBS-injected honey bees were tested at days 0 (A) and 3 (B) after injection. The number of honeybees tested at day 0 post-injection is 60 for each group; the number of honeybees tested at day 3 for IAPV-injected group is 14 and 29 for the PBS-injected group (*=p<0.05, **=p<0.01); sucrose responsiveness of honey bees injected with IAPV with 1: 200 dilution and PBS-injected honey bees tested at days 0 (C) and 2 (D) after injection. The number of honey bees tested at day 0 post-injection is 60 for each group; the number of honey bees tested at day 2 for IAPV-injected group is 22 and 30 for the PBS-injected group (*=p<0.05, **=p<0.01).

 The honey bees infected with 1 ul of IAPV (1: 500 dilution) containing approximately 44 copies of IAPV showed similar sucrose responsiveness at days 0, 1 and 2 after injection in comparison to the sham-injected group of honey bees (Figure 2A). However, significant differences in responsiveness to the low sucrose concentrations between the two groups were found at day 3 after injection (Fisher exact test, p<0.05). These honey bees were found to be more responsive to low sucrose concentrations than that of the sham-injected group of honey bees, and they showed no difference in responsiveness to the high sucrose water (Figure 2B).

 There was no difference regarding sucrose responsiveness between honey bees infected with 1 ul of IAPV (1: 200 dilution) containing approximately 110 copies of IAPV and honey bees injected with PBS at days 0 and 1 after injection (Figure 2C). However, foragers infected with IAPV exhibited significantly higher responsiveness to the low sucrose water than that of foragers injected with PBS at day 2 after injection (Fisher exact test, p<0.05), and they showed no difference in responsiveness to the high sucrose water (Figure 2D). 

### Survival rates of inoculated honey bees

**Figure 3:**
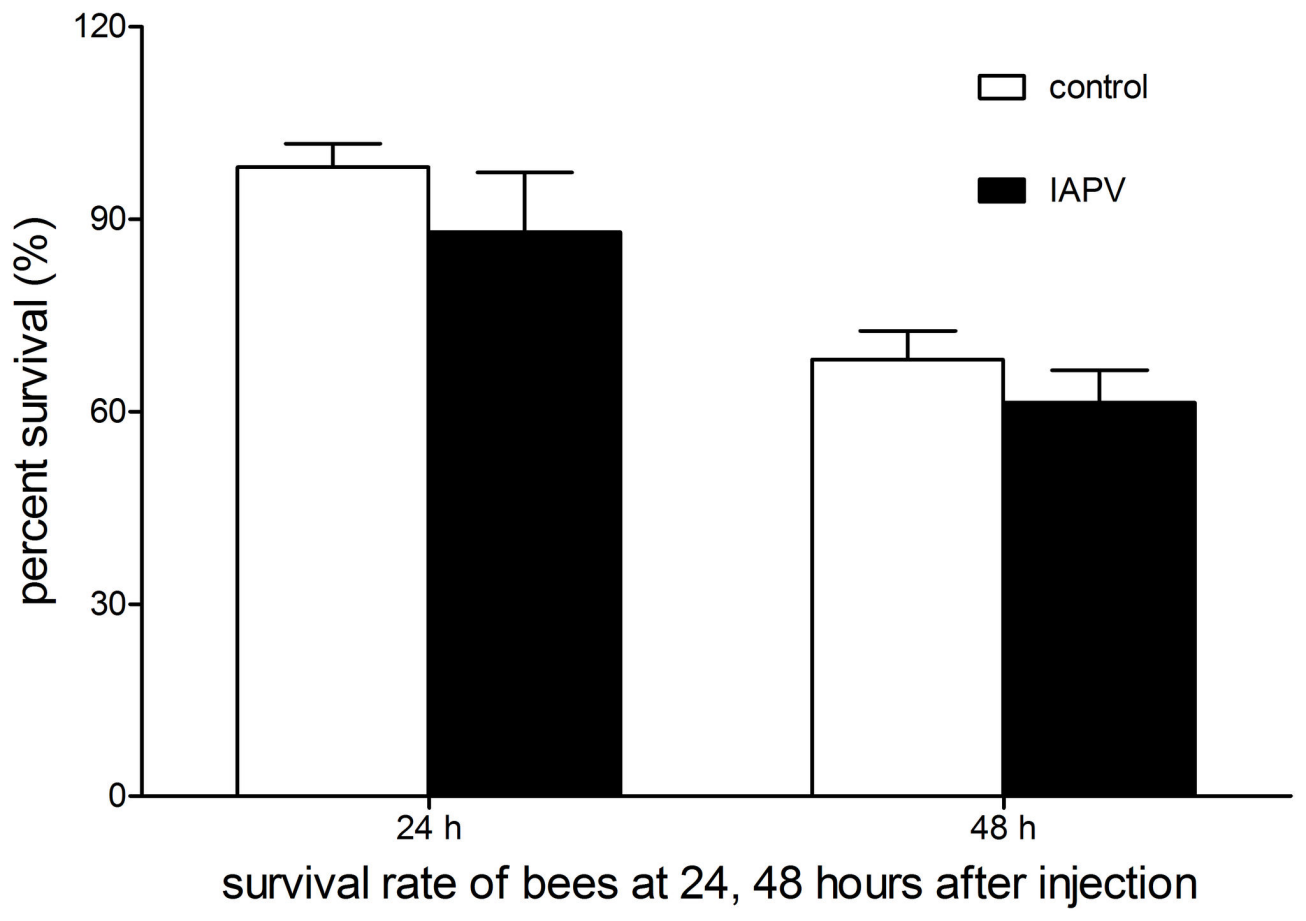
Analysis of survival rates. Comparison of survival rates between honey bees injected with IAPV and honey bees of the control groups at 24, 48 hours after injection. The data are expressed as mean ± SD of four independent experiments.

There was no significant difference between honey bees injected with IAPV and the control group in survival rates (Figure 3). The proportion of live honey bees to dead honey bees was 98.1% and 88.0% for the control group and IAPV-infected honey bees at 24 hours after injection (independent samples t-test, t=2.025, df=3.933, p>0.05), and the proportion at 48 hours after injection was 68.1% and 61.4% for the two groups (independent samples t-test, t=2.012, df=6, p>0.05).

### Detection of IAPV in heads of honey bees

**Figure 4:**
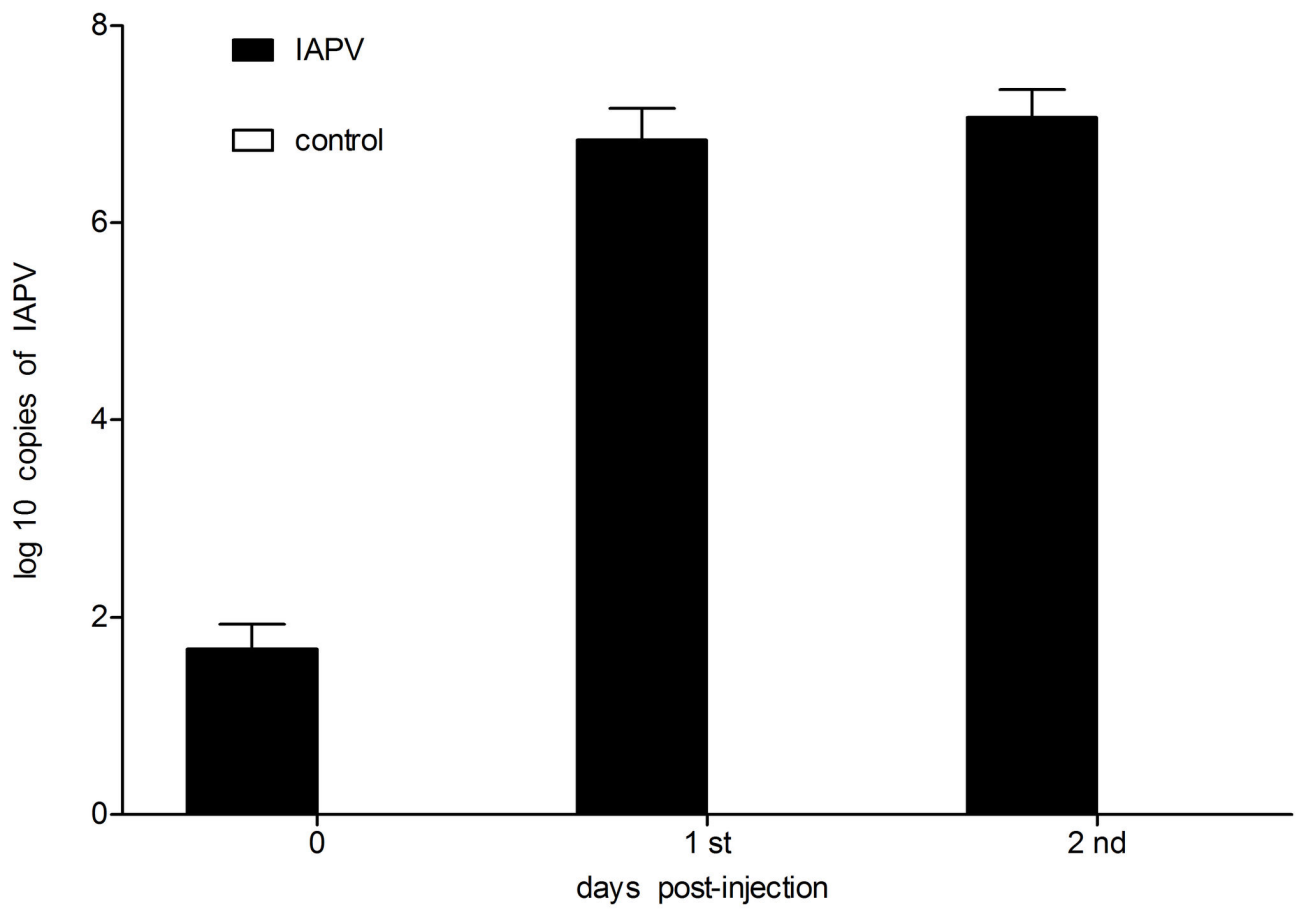
The exact number of copies of IAPV in heads of honey bees. The exact number of copies of IAPV in heads of honey bees collected at day 0, day 1 and day 2 after injections with 1 ul of IAPV (1: 200 dilution) and 1 ul of PBS respectively. Error bars show SD.

 The exact number of copies of IAPV in heads of honey bees was shown in Figure 4 by relating the Ct values to the standard curve (Figure S1). There were about 48 copies of IAPV in the heads of honey bees at day 0 post-injection with IAPV; 6.9x10^6^ IAPV copies were detected in the heads of honey bees at day 1 post-injection, and the number of IAPV copies in the heads of honey bees at day 2 post-injection was 1.2x10^7^. IAPV was not detected in the heads of honey bees injected with PBS. The number of copies of IAPV increased dramatically at day 1 post-injection, however, the IAPV load exhibited no obvious increase in heads of honey bees at day 2 post-injection compared to day 1 post-injection. 

### Impairment of the homing ability of IAPV-infected honey bees

**Figure 5:**
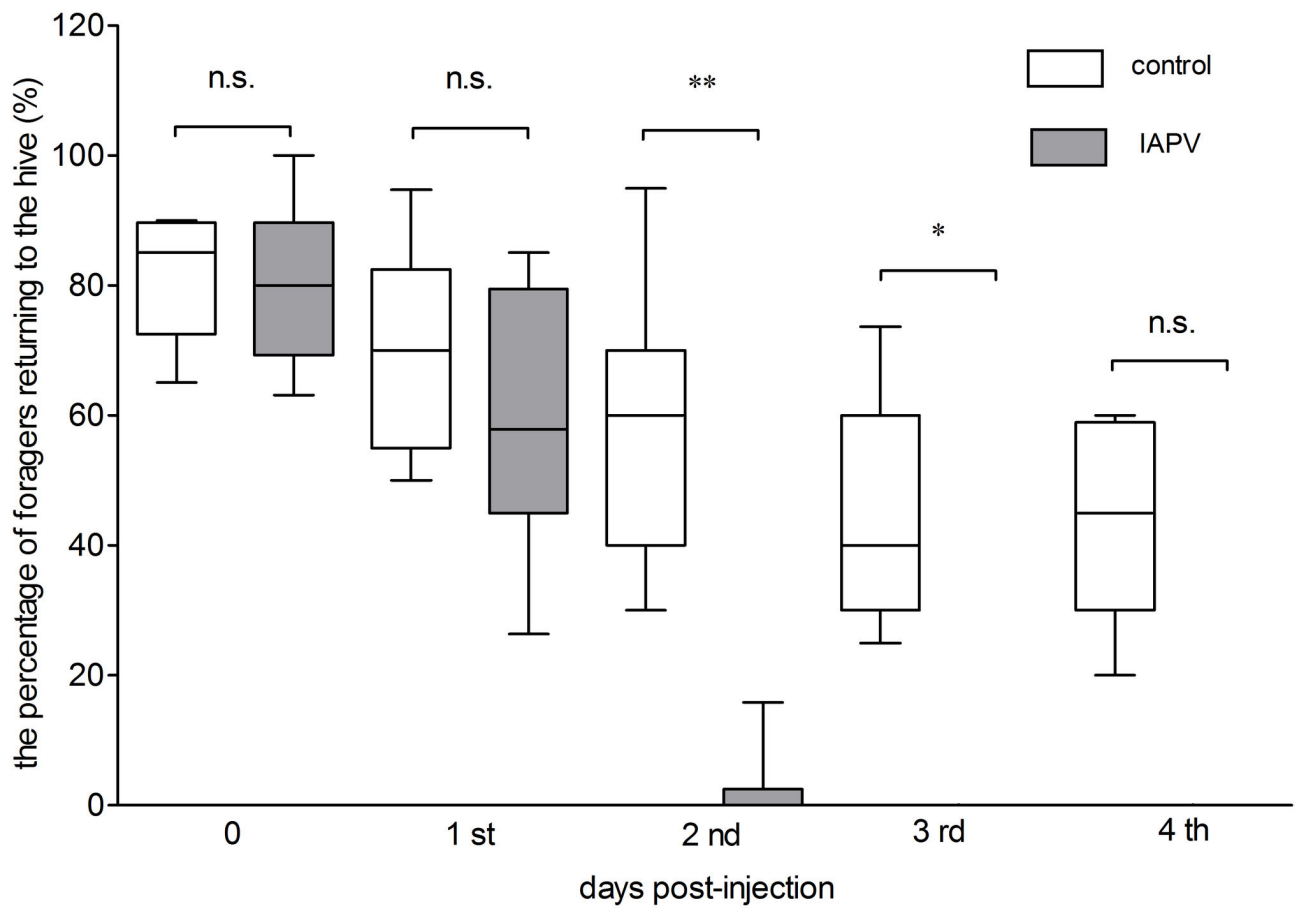
Effects of IAPV on the homing ability of forager honey bees. The abscissa represents days after foragers were injected with PBS and IAPV respectively. The ordinate represents the percentage of foragers departing and returning to the hive per day. *=p<0.05, **=p<0.01, n.s.= not significant. Error bars show SEM.

 The number of honey bees equipped with tags for control animals and IAPV-infected honey bees was 180, respectively. Only 4 honey bees (1.1%) failed to take off within 5 minutes and these honey bees were excluded from analysis. The homing ability of foragers infected with IAPV was depressed significantly in comparison to foragers injected with PBS (Figure 5). There was no significant difference (Kruskal-Wallis test followed by Dunn's multiple comparison test, p>0.05) between the two groups in the percentage of foragers returning to the hive at days 0, 1 and 4 post-injection. However, significant differences were found at 2 (p<0.01) and 3 (p<0.05) days post-injection between the two groups. The percentage of returning foragers injected with PBS returning at days 0, 1, 2 and 3 post-injection was 81.0%, 68.9%, 58.7% and 46.0%. For the IAPV injection group, the percentage was 79.6%, 59.2% , 2.3% and 0%, respectively.

 At day 2 post-injection, there were around 12 foragers injected with PBS returning to the hive compared with almost no IAPV-injected foragers returning to the hive. About 10 foragers were departing and returning to the hive 3 and 4 days after being injected with PBS, however, there were no foragers departing and returning at day 3 or day 4 after being injected with IAPV. Similar results were obtained from three independent experiments and the data provided clear evidence that a viral infection of IAPV in the heads may make honey bees lose their way back to the hive.

## Discussion

Previous studies showed that DWV impaired the sucrose responsiveness of pollen foragers [21]. Our results demonstrated that IAPV also affected the sucrose responsiveness of pollen foragers, causing the honey bees to be more responsive to low concentrations of sugar water as a result of that metabolic stress, compared to honey bees without IAPV infection. IAPV and DWV are both common honey bee viruses and may affect behaviors of honey bees in the same way. However, the underlying disease mechanisms of the virus infections warrants further investigation. Additionally, our study of honey bees infected with different concentrations of viral particles showed similar changing patterns towards different concentrations of sucrose solutions. When honey bees were injected with higher initial concentrations of virus particles, their higher responsiveness to low concentrations of sucrose solutions could be aroused in advance compared to honey bees injected with lower initial concentrations of virus particles. Besides, a higher percentage of honey bees showing dull responses and exhibiting small movements of the proboscis was found in IAPV-infected honey bees after 2 days infection compared to control animals. As a result, these honey bees were not used for data collection. So, the number of IAPV-infected honey bees used for data collection was therefore limited in comparison to control animals.

 We found that sucrose responsiveness in control animals vary between days in the same group (Figure 2), and a similar phenomenon was also found in previous research [21]. Given that PBS was injected into the hemolymph of the honey bees using a needle that may impose some extra stress on the honey bees and the extra stress may be the reason why the sucrose sensitivity of control animals was not consistent between days. In addition, sucrose responsiveness of one group of honey bees was tested in early September 2011 (Figure 2A, B), and sucrose sensitivity of another group of honey bees was tested in mid April 2012 (Figure 2C, D). The variation of sucrose sensitivity in control animals between the two groups may be due to the differential weather and floral resources between the two seasons [48]. 

 Foragers infested with *Nosema* sp. or infested with *V. destructor* showed a lower return rate compared to that of non-infested foragers, which was explained as a common response to pathogens responsible for honey bee diseases [29,30]. Bortolotti et al. reported that honey bees treated with sub-lethal doses of imidacloprid did not return to the hive and showed decreased foraging activity compared to that of the control [49]. Henry et al. also reported that the homing ability of forager honey bees was impaired by thiamethoxam intoxication using RFID systems [28]. Here, we provided additional evidence that IAPV infection could impair the homing ability of honey bee foragers as well. Because there was no significant difference between IAPV-infected honey bees and honey bees of the control groups in survival rates after 24 and 48 hours post-injection, we can exclude the possibility that the honey bees that were not returning to the hive in the evening at day 2 post-injection (approximately after 48 hours injection) were primarily due to death caused by mechanism damage. In fact, honey bees that were infected with IAPV at day 2 post-injection departed from the hive in the morning and some honey bees also foraged normally during the daylight hours, however, they did not return to the hive in the evening and they may have gotten lost and died later in the field before returning to the hive. There were also no dead honey bees found in and outside the hives during the following days. PBS treated honey bees, however, foraged every day, albeit, with the gradual reduction in the number of honey bees returning to the hive during the following days even eighteen days after injection.

 A limited number of IAPV copies were detected in the heads of foragers injected with IAPV at day 0 post-injection, and the number of IAPV copies increased rapidly in the heads of foragers on day 1 post-injection. However, the data also show little difference regarding the number of IAPV copies between day 1 post-injection and day 2 post-injection in the heads of foragers injected with IAPV. We concluded that IAPV may interfere with normal nervous system functions in the brains of honey bees and cause foragers to lose their way back to the hives based on the homing behaviors of IAPV-injected foragers and the number of IAPV copies detected in the heads of honey bees. In addition, detection of the viral infection in the heads of honey bees from commercial apiaries was rare, based on our preliminary experiments. Previous studies revealed that the detection of DWV in honey bees’ heads is rare and represents an overt DWV infection which is also a significant indicator for colony loss associated with DWV infection [44,50,51]. Given the fact that there were about 6.9x10^6^ and 1.2x10^7^ IAPV copies detected in the heads of honey bees at days 1 and day 2 post-injection, respectively, we propose that the high levels of IAPV replication might lead to severe disease in infected honey bees, which lost their navigational abilities and were unable to return to the hives. 

 An impressive result was found during a homing experiment carried out on a rainy day. There were 14 and 13 foragers for IAPV-injected and PBS-injected control group at day 1 post-injection respectively. All the IAPV-injected foragers that foraged on that day did not return to the hive in the evening. However, only 5 PBS-injected foragers foraged on that day, and 4 of those foragers returned to the hive in the evening. Therefore, 12 PBS-injected foragers foraged outside while the number of IAPV-injected foragers was 0 at day 2 post-injection (a sunny day). Foragers infected with IAPV foraged more actively in adverse weather conditions than the sham-injected foragers did, however, they did not return to the hive. A similar phenomenon was also found in honey bees infected by *Nosema* sp., which was interpreted, in part, as compensation for foraging yield because of their shortened lifespan [30]. Regardless, forager honey bees infected with IAPV at day 2 post-injection showed a trend of foraging earlier in the morning compared to that of the control group. Previous studies showed that foragers with lower response thresholds collect lower concentrations of nectar than those with higher response thresholds [52,53], which can be used to explain why foragers infected with IAPV departed the colony earlier in the day and also foraged on the rainy day.

 Our studies clearly showed that both sucrose responsiveness and homing ability of forager honey bees were affected by IAPV which was injected into the hemolymph of honey bees using a 5 ul microsyringe. The wound and stress caused by the needle might simulate the process of a mite's bite. So, it is conceivable that the stress caused by the needle might have some subtle effects on the honey bees. We can’t rule out the possibility that microsyringes used to administer injections might impose additional stress on the honey bees, but it is an effective method to infect the viruses in honey bees [21]. Sucrose responsiveness reflects the division of foraging labor of honey bees [54] and homing involves spatial memory and navigation of honey bees [26,27]. Viral infection in heads may cause disorders in foraging roles of honey bees, with honey bees foraging abnormally, and may enable the virus to interfere with brain functions that are responsible for navigation, orientation and spatial memory in the honey bees. After foraging, IAPV-infected honey bees initiating homing flight from the foraging site to the hive may lose their way back to the hive due to loss in spatial memory. Our results provided first evidence that viral infection in the heads of honey bees could impair the homing ability of forager honey bees. This study is in line with previous studies that sublethal dosages of insecticides (imidacloprid, thiamethoxam et al.) could affect homing ability and foraging activity of honey bees [28,33,49,55]. Colony losses reported worldwide in recent years can, therefore, be triggered in part by multiple stressors including insecticides and viruses. 

## Supporting Information

Figure S1 **The standard curve for IAPV obtained using SYBR Green qPCR and serial diluted plasmid as template.**
(TIF)
