# Supplementary figures and images for: Viral Infection Affects Sucrose Responsiveness and Homing Ability of Forager Honey Bees, Apis mellifera L
Source: PLoS One. 2013 Oct 10;8(10):e77354. doi: 10.1371/journal.pone.0077354 (PMC3795060; doi:10.1371/journal.pone.0077354)

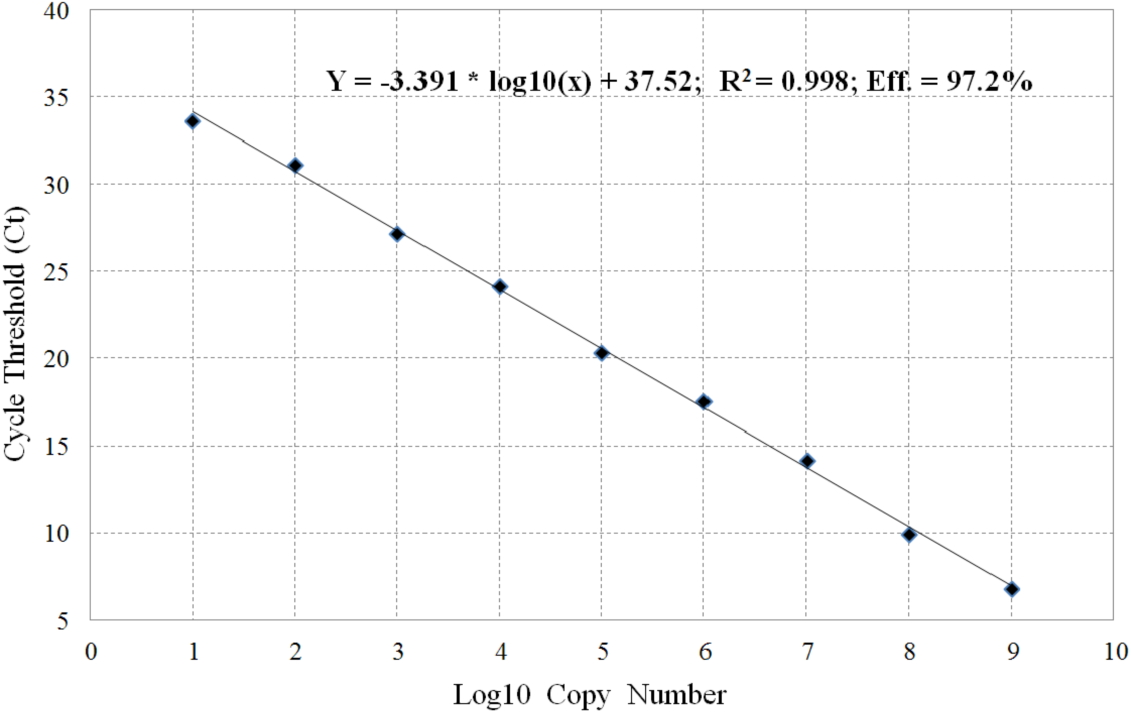

Supplement: Figure S1 — The standard curve for IAPV obtained using SYBR Green qPCR and serial diluted plasmid as template. (TIF) [file pone.0077354.s001.tif]
